# Phenolics Profile and Antioxidant Activity Analysis of Kiwi Berry (*Actinidia arguta*) Flesh and Peel Extracts From Four Regions in China

**DOI:** 10.3389/fpls.2021.689038

**Published:** 2021-07-01

**Authors:** Jiyue Zhang, Ningxuan Gao, Chi Shu, Shunchang Cheng, Xiyun Sun, Changjiang Liu, Guang Xin, Bin Li, Jinlong Tian

**Affiliations:** Key Laboratory of Healthy Food Nutrition and Innovative Manufacturing of Liaoning Province, National R&D Professional Center for Berry Processing, College of Food Science, Shenyang Agricultural University, Shenyang, China

**Keywords:** kiwi berry, peel, phenolic acid, vitamin C, antioxidant activity

## Abstract

The kiwi berry (*Actinidia arguta*) has been widely studied because of its rich phenolic, flavonoid, and vitamin C contents. Numerous reports have demonstrated that fruit peels contain higher phenolic content and antioxidant activity than that of flesh. In this study, the phytochemical content and antioxidant activities of peel and flesh extracts of six kiwi berries were analyzed from four regions (namely, Dandong, Benxi, Taian, and Tonghua) in China. The antioxidant activity was determined using the peroxyl radical scavenging capacity (PSC) and cellular antioxidant activity (CAA) assays. The phenolic, flavonoid, and vitamin C contents of kiwi berry peel were 10.77, 13.09, and 10.38 times richer than that of kiwi berry flesh, respectively. In addition, the PSC and CAA values of kiwi berry peel were higher than those of kiwi berry flesh. The analysis of the separation and contents of phenolics were performed by the high-performance liquid chromatography (HPLC)-diode-array detectormass spectrometry/mass (DAD-MS/MS) system, and the results illustrated that protocatechuic acid, caffeic acid, chlorogenic acid, and quinic acid were the major phenolic compounds. In conclusion, this study indicated that kiwi berry peel contains a rich source of antioxidants. These data are of great significance for the full development and utilization of kiwi berries in these four regions of China to produce nutraceutical and functional foods.

## Introduction

The consumption of fruits and vegetables has been encouraged because they are an excellent source of biologically active compounds, including phenolics, carotenoids, tocopherols, and anthocyanins (Celestino and Font, 2020). Increasing the intake of fruit and vegetable significantly reduces the risk of cancer (Fielding and Walsh, 1994), coronary heart disease, and other chronic diseases (Joshipura, 2001).

According to the recent revision of taxonomy, *Actinidia* is a member of the Actinidiaceae family and it contains 54 species (Huang et al., 2004). Among the various types of *Actinidia*, kiwi berry (*Actinidia arguta*) is a relatively new type of commercially grown fruit, which is called the “mini kiwi” and “baby kiwi” (Williams et al., 2003). Kiwi berry possesses delicious taste and health-promoting properties (Ferguson and Ferguson, 2003). In addition, kiwi berries are extremely abundant in phenolics (Fisk et al., 2006), flavonoids (Park et al., 2014), vitamin C (Latocha, 2015), carotenoids, chlorophylls (Nishiyama et al., 2005), proteins, and minerals (Bieniek and Dragańska, 2013; Latocha, 2015). Phenolics reduce the risk of many chronic diseases (Liu, 2013). In addition, flavonoids have anti-inflammatory, anti-allergic, anticarcinogenic, and anti-ulcer properties (Nayak et al., 2011). Vitamin C is considered the most important vitamin because of its significant antioxidant activity (Forastiere et al., 2000). In this study, both phenolic and flavonoid contents were determined using the Liu’s method (He et al., 2008; Guo et al., 2012). The vitamin C content was measured using the high-performance liquid chromatography (HPLC) analysis (Mekky et al., 2018). The analysis of the separation and contents of phenolics were performed by the HPLC-DAD-MS/MS system (Lang et al., 2020). The phenolics profile and antioxidant activities of fruits are different, and fruits with high antioxidant activity usually contain more antioxidants (Guo et al., 1997). It is worth noting that the peel and seed extracts of some fruits have higher antioxidant activities than those of flesh extracts (Sun et al., 2021); mango peel is a rich source of phenolics, carotenoids, and anthocyanins (Ajila et al., 2007b). In contrast, the skin of the kiwi berry is mostly smooth and without hair, which makes the whole fruit suitable for direct consumption without removing the skin (Jackson and Harker, 1997). The intake of antioxidants in kiwi berries is nearly three times higher than that in kiwifruit (Horák et al., 2019). Previous study has reported the phenolics, ascorbate, and antioxidant potency of peel and flesh extracts of kiwi berry (Latocha, 2015); however, the peroxyl radical scavenging capacity (PSC) and cellular antioxidant activity (CAA) have not yet been investigated.

To gain insight into the composition and antioxidant activities of kiwi berry flesh and peel extracts cultivated in four regions of China (namely, Dandong, Benxi, Taian, and Tonghua), the objectives of this study were (1) to determine the total phenolic content (TPC), total flavonoid content (TFC), and vitamin C content of free and bound fractions of flesh and peel extracts; (2) to identify and quantify the free and bound phenolic contents of flesh and peel extracts; and (3) to determine the PSC and CAA values of free and bound fractions of flesh and peel extracts of six common kiwi berry varieties. Since peels are not currently used for commercial purposes, they are discarded as waste and become a source of pollution (Vicenssuto and Castro, 2020). This study of the phenolics profile and antioxidant activities of commercial kiwi berry cultivars in China concluded that kiwi berry peel extract had more potential than flesh extract as a health supplement rich in natural antioxidants and deserves further research.

## Materials and Methods

### Chemicals and Reagents

Ascorbic acid, gallic acid, Folin–Ciocalteu reagent, sodium borohydride (NaBH_4_), aluminum chloride, chloranil, vanillin, and 2,7-dichlorodihydrofluorescein diacetate (DCFH-DA) were obtained from Sigma Chemical Co (St. Louis, MO, United States). Potassium hydroxide (KOH), 2,20-azobis-amidinopropane (ABAP), sodium hydroxide (NaOH), potassium dihydrogen phosphate (KH_2_PO_4_), potassium hydrogen phosphate (K_2_HPO_4_), and sodium bicarbonate (NaHCO_3_) were obtained from Aladdin Co., Ltd (Shanghai, China). All reagents used in this study were of analytical grade. Methanol and acetonitrile used for the HPLC analysis were purchased from Aladdin Co., Ltd (Shanghai, China).

### Sample Preparation

Kiwi berries generally germinate in mid-April, enter the peak growth period from late May to mid-June, enter the full bloom period in late June, and mature around August–October. Therefore, from August to October, kiwi berries in most areas of China reach commercial maturity. In this study, kiwi berries were collected from Dandong (LD-241, LD-121), Benxi (Huairou), Taian (Changjiangyihao), and Tonghua (Longcheng, Liaofeng) in China at the commercial maturity stage.

More than 50 fruits of nearly the same size without any disease or pest damage were randomly collected for each variety from four different regions. The samples were placed in cooler containers and immediately transported to the laboratory. Each kiwi berry variety was randomly divided into three groups (each group approximately 100–150 g) as three replicates for each experiment. Different fruit pieces from each kiwi berry variety were washed, manually peeled, and mixed. After the flesh and peel were separated, the weight of the flesh and peel in relation to the whole fruit, soluble solids, and pH were immediately determined. The remaining samples were frozen at −20°C for not longer than 1 week, until phenolic extraction.

### Extraction of Free and Bound Phenolics

Phenolics were extracted using a previously reported method with some modifications (Gorinstein et al., 2009; Guo et al., 2012). In brief, 100 g of fresh kiwi berry sample was extracted with 100 ml of 80% acetone for 10 min, and the mixture was mashed and then filtered using a filter paper. The filtrate was collected after centrifugation at 2,500 × *g* for 10 min and filtered through a filter paper. All extractions were performed twice. The supernatants were pooled and evaporated at 45°C. The extraction was reconstituted to 20 ml with distilled water to obtain the free phenolic fraction. All residues were collected in centrifuge tubes and digested with 20 ml of 4 N NaOH with shaking for 1 h under nitrogen at room temperature (23°C). The pH of the mixture was adjusted to 2.0 using concentrated HCl and fractioned with ethyl acetate five times. Ethyl acetate was removed by rotary evaporation, and the extraction was reconstituted to 20 ml with distilled water to obtain the bound phenolic fraction. The samples were frozen in liquid nitrogen and stored at −80°C until use.

### Total Soluble Solids (°Brix)

Total soluble solids (TSS) from accurately weighed kiwi berry flesh and peel samples (5 g) were measured using a digital refractometer (Atago Co., Ltd., Tokyo, Japan) and recorded as “degrees Brix” (°Brix), which is equivalent to a percentage (%). The Brix scale or °Brix is numerically equal to the percentage of sugar and other dissolved solids in the solution.

### Total Phenolic Content Determination

Total phenolic content was measured using the colorimetric Folin–Ciocalteu method, as previously reported (Guo et al., 2012). Gallic acid was used as a standard. TPC was expressed as mg of gallic acid equivalents per 100 g fresh weight (mg GAE/100 g FW). The data are reported as the mean ± standard deviation (SD) for three replications.

### Total Flavonoid Content Determination

Total flavonoid content was determined using the sodium borohydride chloranil (SBC) protocol (He et al., 2008). Catechin was used as a standard. TFC was expressed as mg of catechin equivalents per 100 g fresh weight (mg CE/100 g FW). The data are reported as the mean ± SD for three replications.

### Vitamin C Content Determination

The vitamin C content was measured using the HPLC analysis, with some modifications (Mekky et al., 2018). Vitamin C was obtained from 100 g of kiwi berry with a mixture of 1% meta-phosphoric acid and 1% perchloric acid. Liquid chromatography was used to identify and quantify vitamin C (Agilent 1290II-6460). Detection was performed using an Agilent SB-C18 column (2.1 × 100 mm, 1.8 μm). The mobile phase consisted of A (0.1% formic acid–water) and B (0.1% formic acid–acetonitrile). The vitamin C content was expressed as mg per 100 g fresh weight (mg/100 g FW). The data are reported as the mean ± SD for three replications.

### Identification of Phenolic Compounds of Kiwi Berry

The analysis of the separation and contents of phenolics were performed by the HPLC-DAD-MS/MS system (Agilent 1290II-6460), the method suggested in a previous report and was used with some changes (Lang et al., 2020). The chromatographic column was Agilent SB-C18 column (2.1 × 100 mm, 1.8 μm). The chromatographic column temperature was 30°C. Different phenolics of kiwi berry were separated in gradient elution. The mobile phase consisted of A (0.1% formic acid–water) and B (0.1% formic acid–acetonitrile). The flow rate was 0.2 ml/min. The injection volume of samples was 20 μl. The autosampler temperature was the same as room temperature. The gradient elution system was as follows: 0–4 min 10% B; 4–6 min 10–21.5% B; 6–16 min 21.5–28% B; 16–25 min 28–50% B; 25–28 min 50–95% B; and 28–30 min 95–95% B.

Mass spectrum acquisition parameters include ion source type electrospray ionization; ion source temperature, 350°C; negative iron mode; capillary voltage, 3.5 kV; atomizing gas flow, 10 L/min; atomizing gas pressure, 45 psi; mode, MS2 scan; scan time, 300 ms; scan step, 0.1 amu; and fragmentor, 120 V. Software for data acquisition and processing include the automatic integration used Agile 2 integrator software and the mass spectrum extraction used average spectrum of 10% peak height. The real-time chromatogram was collected at 280 nm by the diode array detection. The monomer peaks were determined by the retention time and MS/MS fragments. Chlorogenic acid was used as the standard of phenolic acids, and quercetin-3-*O*-glucoside was used as the standard of flavanols and three flavonols. The content of phenolics was calculated by corresponding peak area and calibration curves of standards.

### Determination of Total Antioxidant Activity Using a Rapid Peroxyl Radical Scavenging Capacity Assay

Antioxidant activities were determined using the PSC method, as previously described (Adom and Liu, 2005). The PSC values were expressed as μmol vitamin C equivalents per 100 g fresh weight (μmol VCE/100 g FW). The data are reported as the mean ± SD for three replications.

### Determination of Total Antioxidant Activity Using a Cellular Antioxidant Activity Assay

The CAA assay was conducted as previously described (Liu, 2007). In this study, HepG2 cells at 20–29 passages were used as a model to evaluate the CAA value of flesh and peel extracts. Quercetin was used as a standard. The CAA values were expressed as μmol of quercetin equivalents per 100 g fresh weight (μmol QE/100 g FW). The data are reported as the mean ± SD for three replications.

### Statistical Analysis

All data are reported as the mean ± standard deviation (SD). A one-way analysis of variance (ANOVA) was performed to determine the overall effect of different treatments, and the Duncan’s test was used for multiple comparisons. All analyses were performed using SPSS software 19.0 (SPSS Inc., Chicago, IL, United States) with a significance level of 0.05 (two-tailed *p* value). To establish a correlation between the phenolics profile and antioxidant activities, the multivariate correlation was conducted by partial least squares regression (PLS) using Unscrambler 10.1 (Camo Process AS, Oslo, Norway). In the PLS method, the predictors (variable *X*) were the content of phenolics profile, with the responses (variable *Y*) being the PSC and CAA values.

## Results

### Kiwi Berry Conventional Quality

The six kiwi berry varieties had different shapes and sizes. The kiwi berry conventional quality of varieties from Dandong, Benxi, Taian, and Tonghua regions are shown in Table 1 and Supplementary Figure 1. Changjiangyihao had the largest weight (14.09 g), and LD-121 had the least weight (5.35 g). The percentages of edible skin in each variety were different. Overall, although the sample date, color, and shape were different, no differences were observed in TSS and pH of the six kiwi berry varieties from the four different regions.

**Table 1:** Kiwi berry conventional quality.^a^

Varieties	Origin	Location	Sampled date	Color	Shape	Whole fruit	Peel	Flesh	Peel participation (%)	Brix°	pH
LD-241	Dandong, LN	124°23^'^E, 40°07^’^N	2018.09.15	green	oblateness	12.52 ± 0.42^b^	1.90 ± 0.04^a^	10.62 ± 0.38^c^	15.17 ± 0.21^c^	20.09 ± 0.63	4.02 ± 0.02
LD-121	Dandong, LN	124°23^'^E, 40°07^’^N	2018.09.15	green	circular	5.35 ± 0.26^e^	0.71 ± 0.05^d^	4.64 ± 0.21^f^	13.33 ± 0.37^d^	20.41 ± 0.09	4.22 ± 0.04
Huairou	Benxi, LN	124°17^'^E, 41°24^’^N	2018.09.13	green	oblateness	9.57 ± 0.19^c^	1.79 ± 0.02^a^	7.77 ± 0.18^d^	18.73 ± 0.29^b^	20.25 ± 0.52	4.05 ± 0.01
Changjiangyihao	Taian, SD	117°06^'^E, 36°11^’^N	2018.09.28	pale green	oblong	14.09 ± 0.34^a^	1.59 ± 0.1^b^	12.50 ± 0.21^a^	11.27 ± 0.76^e^	19.94 ± 0.85	4.11 ± 0.04
Longcheng	Tonghua, JL	125°93^'^E, 41°73^’^N	2018.10.01	dark green	elliptic sex	12.78 ± 0.36^b^	0.97 ± 0.05^c^	11.81 ± 0.38^b^	7.60 ± 0.50^f^	20.12 ± 0.07	4.23 ± 0.08
Liaofeng	Tonghua, JL	125°93^'^E, 41°73^’^N	2018.10.01	green	oblateness	8.58 ± 0.38^d^	1.82 ± 0.02^a^	6.75 ± 0.37^e^	21.30 ± 0.86^a^	19.98 ± 0.32	4.21 ± 0.05

a Results are expressed as mean ± SD (*n* = 3). Values of each column with no letters in common are significantly different (*p* < 0.05). Absence of superscript indicates no significant differences.

### Total Phenolic Content of Kiwi Berry

**Figure 1:**
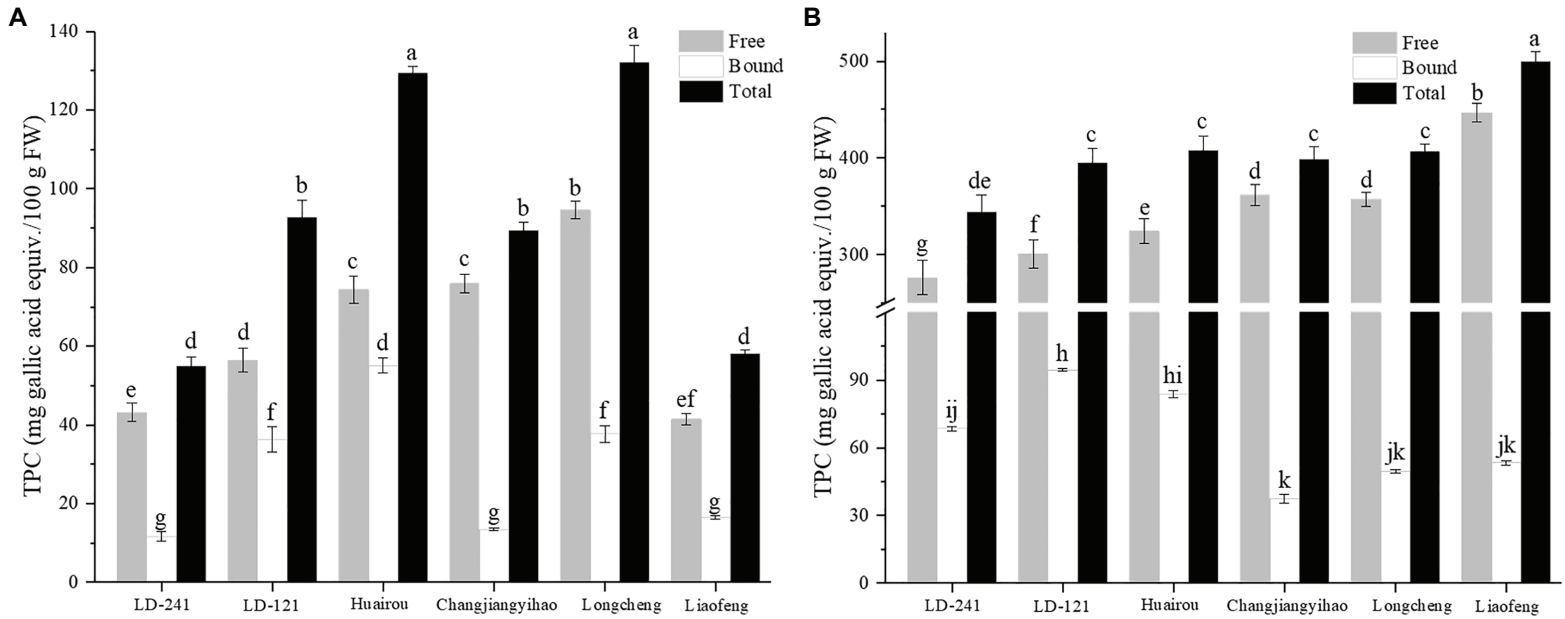
Total phenolic content of flesh extracts **(A)** and peel extracts **(B)** of six kiwi berry varieties (mean ± SD; *n* = 3). Bars with different letters differ significantly (*p* < 0.05).

The TPC of the flesh (Figure 1A) and peel flesh (Figure 1B) extracts of six kiwi berry varieties are shown in Figure 1. The free-TPC was significantly higher than that of the corresponding bound-TPC in both the flesh and peel extracts. As shown in Figure 1A, the free-TPC of flesh extracts ranges from 41.47 mg GAE/100 g FW (Liaofeng) to 94.57 mg GAE/100 g FW (Longcheng), and the bound-TPC of flesh extracts ranges from 11.67 mg GAE/100 g FW (LD-241) to 55.08 mg GAE/100 g FW (Huairou). As shown in Figure 1B, the free-TPC of peel extracts ranges from 276.31 mg GAE/100 g FW (LD-241) to 446.74 mg GAE/100 g FW (Liaofeng); whereas, the bound-TPC of peel extracts ranges from 37.30 mg GAE/100 g FW (Changjiangyihao) to 94.45 mg GAE/100 g FW (LD-121). In general, the peel TPC was considerably higher than that of the corresponding content in the flesh of all six kiwi berry varieties.

### Total Flavonoid Content of Kiwi Berry

**Figure 2:**
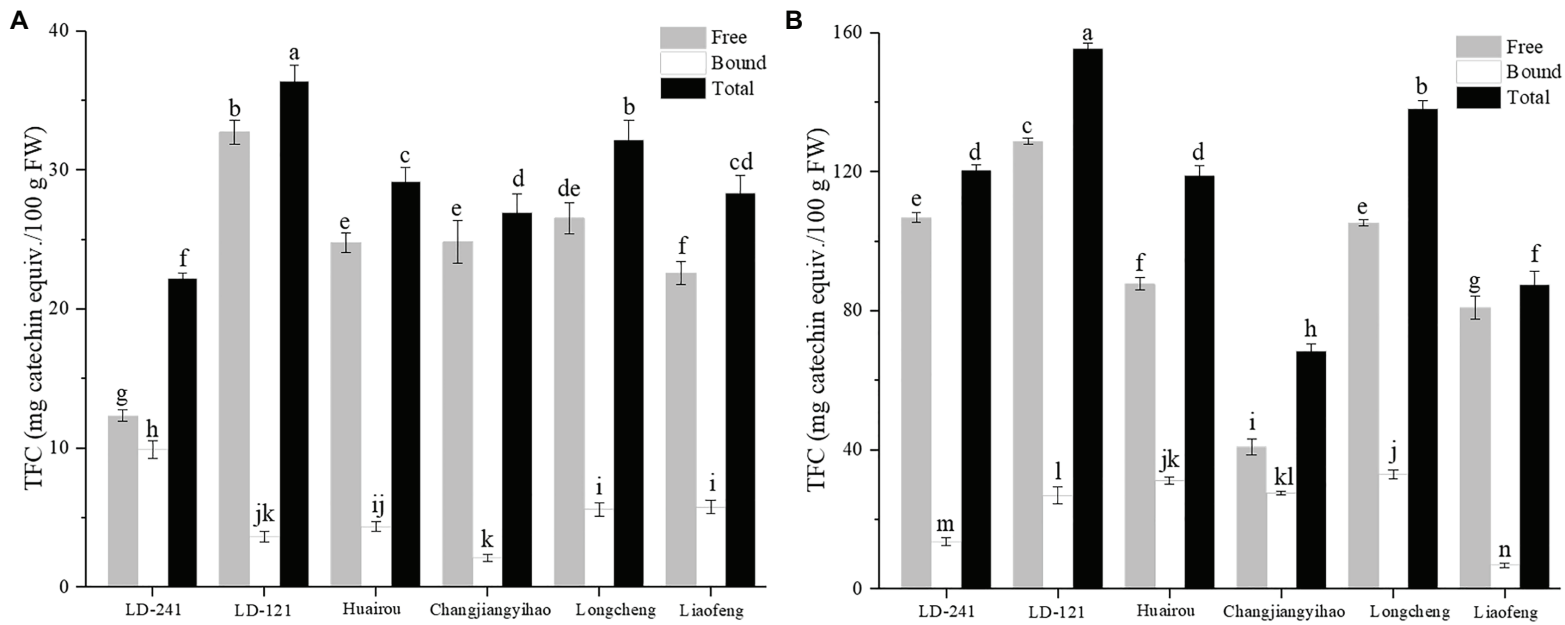
Total flavonoid content of flesh extracts **(A)** and peel extracts **(B)** of six kiwi berry varieties (mean ± SD; *n* = 3). Bars with different letters differ significantly (*p* < 0.05).

The TFC of the flesh (Figure 2A) and peel (Figure 2B) extracts of six kiwi berry varieties is shown in Figure 2. The free-TFC was significantly higher than that of the corresponding bound-TFC in both the flesh and peel extracts. As shown in Figure 2A, the average of free-TFC of flesh extracts is 23.95 mg CE/100 g FW; whereas, the average of bound-TFC of flesh extracts is 5.22 mg CE/100 g FW. As shown in Figure 2B, the average of free-TFC of peel extracts is 91.69 mg CE/100 g FW, and the average of bound-TFC of peel extracts is 23.13 mg CE/100 g FW. The total-TFC of flesh extracts ranged from 22.23 mg CE/100 g FW (LD-241) to 36.34 mg CE/100 g FW (LD-121). The total-TFC of peel extracts ranged from 68.38 mg CE/100 g FW (Changjiangyihao) to 155.54 mg CE/100 g FW (LD-121). Comparatively, the TFC of peel extracts was considerably higher than that of the corresponding flesh extracts among all six kiwi berry varieties.

### Kiwi Berry Vitamin C Content

The vitamin C content of flesh and peel extracts of the six kiwi berry varieties are shown in Table 2. The vitamin C contents of flesh extracts ranged from 6.82 mg/100 g FW (LD-121) to 25.67 mg/100 g FW (Longcheng). The average vitamin C content of flesh extracts was 15.63 mg/100 g FW and varied by 3.76-fold among the six varieties. The vitamin C content of peel extracts ranged from 56.36 mg/100 g FW (LD-241) to 102.07 mg/100 g FW (Liaofeng). The average vitamin C content of peel extracts was 84.93 mg/100 g FW and varied by 1.81-fold in these varieties. The vitamin C content of peel extracts was considerably higher than that of the corresponding flesh extracts among all six kiwi berry varieties.

**Table 2:** Vitamin C content of different parts of six kiwi berry varieties.^a^

Sample	Vitamin C content (mg/100 g FW)	
	Flesh	Peel
LD-241	12.69 ± 1.48^c^	56.36 ± 0.69^e^
LD-121	6.82 ± 0.38^d^	70.76 ± 4.62^d^
Huairou	12.72 ± 0.46^c^	88.65 ± 1.70^c^
Changjiangyihao	22.30 ± 1.26^b^	92.59 ± 3.93^bc^
Longcheng	25.67 ± 2.90^a^	99.12 ± 5.62^ab^
Liaofeng	13.59 ± 0.72^c^	102.07 ± 0.92^a^

a Results are expressed as mean ± SD (*n* = 3). Values of each column with no letters in common are significantly different (*p* < 0.05).

### Phenolic Composition of Kiwi Berry

**Figure 3:**
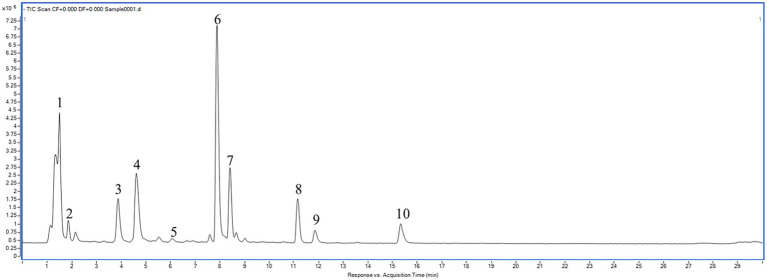
HPLC-DAD-MS chromatogram of kiwi berry phenolic compounds. Peaks: 1, Protocatechuic acid ([M−H]^−^, m/z 153.2; MS/MS, m/z 109.8); 2, Proanthocyanidin B2 ([M−H]^−^, m/z 577.2; MS/MS, m/z 289.1); 3 (+)-gallocatechin ([M−H]^−^, m/z 305.1; MS/MS, m/z 248.1); 4, Quinic acid ([M−H]^−^, m/z 191.1; MS/MS, m/z 146.9); 5, Proanthocyanidin C1 ([M−H]^−^, m/z 865.2; MS/MS, m/z 577.2); 6, Caffeic acid ([M−H]^−^, m/z 179.1; MS/MS, m/z 135.1); 7, Chlorogenic acid ([M−H]^−^, m/z 353.1; MS/MS, m/z 191.1); 8, Quercetin-3-O-galactoside ([M−H]^−^, m/z 463.1; MS/MS, m/z 300.0); 9, Quercetin-3-O-glucoside ([M−H]^−^, m/z 463.1; MS/MS, m/z 301.1); 10, Quercetin-3-O-rutinoside ([M−H]^−^, m/z 609.2; MS/MS, m/z 300.0).

In this study, the composition and content of phenolic monomers were identified and quantified by the HPLC-DAD-MS/MS analysis. As shown in Figure 3, 10 monomers (4 phenolic acids, 3 flavanols, and 3 flavonols) of kiwi berry phenolics were identified. The contents of 10 monomers of the flesh and peel extracts among the 6 kiwi berry varieties are shown in Table 3.

**Table 3:** Phenolic compositions of different parts of six kiwi berry varieties^a^ (μg/g FW).

Phenolics	Free		Bound	
	Flesh	Peel	Flesh	Peel
**Protocatechuic acid**				
LD-241	20.63 ± 1.68^c^	64.53 ± 2.06^a^	8.15 ± 0.01^d^	21.16 ± 0.11^c^
LD-121	26.03 ± 0.98^ab^	56.17 ± 2.72^b^	4.67 ± 0.29^f^	18.04 ± 0.32^d^
Huairou	24.51 ± 0.99^b^	47.32 ± 0.92^c^	9.18 ± 0.06^b^	19.73 ± 0.16^c^
Changjiangyihao	24.98 ± 2.81^b^	55.62 ± 3.20^b^	6.09 ± 0.18^e^	27.75 ± 0.92^b^
Longcheng	28.37 ± 0.25^a^	63.83 ± 2.50^a^	13.04 ± 0.09^a^	32.32 ± 0.90^a^
Liaofeng	29.16 ± 0.02^a^	49.45 ± 1.58^c^	8.63 ± 0.06^c^	28.67 ± 0.92^b^
**Caffeic acid**				
LD-241	28.76 ± 0.94^a^	71.78 ± 1.85^b^	8.49 ± 0.36^b^	22.63 ± 0.62^b^
LD-121	19.27 ± 0.52^b^	51.71 ± 0.70^d^	7.70 ± 0.15^b^	20.40 ± 0.98^c^
Huairou	15.53 ± 2.66^c^	78.31 ± 0.70^a^	12.15 ± 1.08^a^	31.13 ± 1.17^a^
Changjiangyihao	10.67 ± 0.09^d^	62.30 ± 1.53^c^	5.70 ± 0.08^d^	19.45 ± 0.38^c^
Longcheng	11.43 ± 0.41^d^	62.79 ± 2.43^c^	7.37 ± 0.31^bc^	20.55 ± 1.30^c^
Liaofeng	20.48 ± 2.31^b^	59.91 ± 2.29^c^	6.38 ± 0.23^cd^	18.79 ± 0.49^c^
**Chlorogenic acid**				
LD-241	19.45 ± 0.38^c^	45.40 ± 2.32^c^	7.39 ± 0.23^bc^	17.95 ± 0.14^e^
LD-121	24.70 ± 0.52^b^	39.30 ± 0.47^d^	12.70 ± 0.44^a^	21.14 ± 0.68^d^
Huairou	26.15 ± 0.66^a^	61.15 ± 0.33^b^	6.57 ± 0.24^c^	22.60 ± 0.44^c^
Changjiangyihao	17.27 ± 0.36^d^	68.68 ± 1.79^a^	5.34 ± 0.20^d^	30.62 ± 0.46^a^
Longcheng	10.88 ± 0.05^e^	43.05 ± 0.66^c^	3.34 ± 0.95^e^	18.85 ± 0.73^e^
Liaofeng	18.54 ± 0.37^c^	59.16 ± 0.03^b^	8.42 ± 0.34^b^	25.42 ± 0.80^b^
**Quinic acid**				
LD-241	8.44 ± 0.04^c^	37.08 ± 0.44^c^	3.40 ± 0.37^c^	15.59 ± 0.57^c^
LD-121	9.57 ± 0.14^b^	43.67 ± 0.59^b^	4.43 ± 0.32^b^	22.79 ± 1.04^a^
Huairou	12.66 ± 0.17^a^	33.68 ± 1.13^d^	4.44 ± 0.26^b^	12.58 ± 0.42^d^
Changjiangyihao	12.35 ± 0.21^a^	24.23 ± 0.43^e^	5.51 ± 0.30^a^	9.39 ± 0.27^e^
Longcheng	12.82 ± 0.69^a^	41.76 ± 2.26^b^	5.11 ± 0.10^a^	22.12 ± 1.28^a^
Liaofeng	7.67 ± 0.02^d^	51.24 ± 1.23^a^	2.78 ± 0.08^d^	18.60 ± 0.43^b^
**(+)-gallocatechin**				
LD-241	3.72 ± 0.18^c^	9.24 ± 0.36^d^	1.41 ± 0.27	4.25 ± 0.48
LD-121	2.21 ± 0.07^e^	8.66 ± 0.15^d^	0.20 ± 0.02	2.46 ± 0.27
Huairou	3.62 ± 0.16^c^	13.73 ± 0.05^b^	ND^b^	ND
Changjiangyihao	4.36 ± 0.13^b^	14.52 ± 0.31^a^	ND	ND
Longcheng	6.23 ± 0.03^a^	11.60 ± 0.42^c^	ND	ND
Liaofeng	2.88 ± 0.13^d^	6.35 ± 0.31^e^	ND	ND
**Proanthocyanidin B2**				
LD-241	3.01 ± 0.32^b^	5.40 ± 0.36^bc^	ND	ND
LD-121	1.82 ± 0.02^d^	2.95 ± 0.02^d^	ND	ND
Huairou	5.77 ± 0.11^a^	9.59 ± 0.17^a^	ND	ND
Changjiangyihao	2.35 ± 0.03^c^	9.49 ± 0.29^a^	ND	ND
Longcheng	1.50 ± 0.38^d^	4.96 ± 0.03^c^	ND	ND
Liaofeng	2.46 ± 0.03^c^	5.60 ± 0.12^b^	ND	ND
**Proanthocyanidin C1**				
LD-241	2.57 ± 0.39^a^	3.60 ± 0.41^a^	ND	ND
LD-121	2.24 ± 0.08^ab^	2.71 ± 0.09^cd^	ND	ND
Huairou	0.32 ± 0.05^d^	2.44 ± 0.04^d^	ND	ND
Changjiangyihao	0.94 ± 0.03^c^	2.91 ± 0.03^bc^	ND	ND
Longcheng	0.62 ± 0.01^cd^	3.15 ± 0.01^b^	ND	ND
Liaofeng	1.92 ± 0.06^b^	3.92 ± 0.02^a^	ND	ND
**Quercetin-3-O-glucoside**				
LD-241	3.56 ± 0.22^b^	16.57 ± 1.10^a^	ND	ND
LD-121	2.56 ± 0.09^c^	8.58 ± 0.26^c^	ND	ND
Huairou	3.66 ± 0.04^b^	8.40 ± 0.26^c^	ND	ND
Changjiangyihao	3.40 ± 0.09^b^	6.18 ± 0.12^d^	ND	ND
Longcheng	1.64 ± 0.09^d^	4.62 ± 0.04^d^	ND	ND
Liaofeng	4.79 ± 0.21^a^	12.18 ± 1.51^b^	ND	ND
**Quercetin-3-O-rutinoside**				
LD-241	5.32 ± 0.03^a^	19.26 ± 1.45^a^	ND	ND
LD-121	2.52 ± 0.16^d^	5.51 ± 0.05^d^	ND	ND
Huairou	3.65 ± 0.11^c^	14.27 ± 0.45^b^	ND	ND
Changjiangyihao	2.68 ± 0.21^d^	15.25 ± 0.40^b^	ND	ND
Longcheng	5.52 ± 0.23^a^	20.08 ± 1.46^a^	ND	ND
Liaofeng	4.55 ± 0.03^b^	9.54 ± 0.23^c^	ND	ND
**Quercetin-3-O-galactoside**				
LD-241	5.48 ± 0.17^b^	22.35 ± 1.34^a^	ND	ND
LD-121	2.34 ± 0.03^e^	12.48 ± 1.84^b^	ND	ND
Huairou	1.88 ± 0.07^f^	13.45 ± 0.06^b^	ND	ND
Changjiangyihao	6.14 ± 0.05^a^	22.70 ± 3.85^a^	ND	ND
Longcheng	2.77 ± 0.05^d^	19.30 ± 1.30^a^	ND	ND
Liaofeng	3.50 ± 0.37^c^	20.51 ± 1.32^a^	ND	ND

a Results are expressed as mean ± SD. Values of each column with no letters in common are significantly different (*p* < 0.05).

b ND = not detected.

Four phenolic acids, i.e., protocatechuic acid, caffeic acid, chlorogenic acid, and quinic acid, were the predominant phenolics in kiwi berry. The free phenolic acid contents were significantly higher than the corresponding bound phenolic acid contents both in the flesh and peel extracts. The average of free protocatechuic acid contents of flesh extracts was 25.61 μg/g FW. The average of bound protocatechuic acid contents of flesh extracts was 8.29 μg/g FW. The total protocatechuic acid contents of flesh extracts ranged from 28.77 μg/g FW (LD-241) to 41.41 μg/g FW (Longcheng). The average of free protocatechuic acid contents of peel extracts was 56.15 μg/g FW. The average of bound protocatechuic acid contents of peel extracts was 24.61 μg/g FW. The total protocatechuic acid contents of peel extracts ranged from 67.05 μg/g FW (Huairou) to 96.16 μg/g FW (Longcheng). The average of free caffeic acid contents of flesh extracts was 17.69 μg/g FW. The average of bound caffeic acid contents of flesh extracts was 7.97 μg/g FW. The total caffeic acid contents of flesh extracts ranged from 16.37 μg/g FW (Changjiangyihao) to 37.25 μg/g FW (LD-241). The average of free caffeic acid contents of peel extracts was 64.47 μg/g FW. The average of bound caffeic acid contents of peel extracts was 22.16 μg/g FW. The total caffeic acid contents of peel extracts ranged from 72.12 μg/g FW (LD-121) to 109.44 μg/g FW (Huairou). The average of free chlorogenic acid contents of flesh extracts was 19.50 μg/g FW. The average of bound chlorogenic acid contents of flesh extracts was 7.29 μg/g FW. The total chlorogenic acid contents of flesh extracts ranged from 14.23 μg/g FW (Longcheng) to 37.40 μg/g FW (LD-121). The average of free chlorogenic acid contents of peel extracts was 52.79 μg/g FW. The average of bound chlorogenic acid contents of peel extracts was 22.76 μg/g FW. The total chlorogenic acid contents of peel extracts ranged from 60.44 μg/g FW (LD-121) to 99.30 μg/g FW (Changjiangyihao). The average of free quinic acid contents of flesh extracts was 10.58 μg/g FW. The average of bound quinic acid contents of flesh extracts was 4.28 μg/g FW. The total quinic acid contents of flesh extracts ranged from 10.45 μg/g FW (Liaofeng) to 17.93 μg/g FW (Longcheng). The average of free quinic acid contents of peel extracts was 38.61 μg/g FW. The average of bound quinic acid contents of peel extracts was 16.84 μg/g FW. The total quinic acid contents of peel extracts ranged from 33.62 μg/g FW (Changjiangyihao) to 69.84 μg/g FW (Liaofeng). The phenolic acid contents of the peel extracts were considerably higher than those of corresponding flesh extracts among all six kiwi berry varieties.

Three flavanols, i.e., (+)-gallocatechin, proanthocyanidin B2, and proanthocyanidin C1, were also found in kiwi berry. Bound (+)-gallocatechin were only found in LD-241 and LD-121. Proanthocyanidin B2 and proanthocyanidin C1 were only found in free fractions. The free (+)-gallocatechin contents of flesh extracts ranged from 2.21 μg/g FW (LD-121) to 6.23 μg/g FW (Longcheng). The free (+)-gallocatechin contents of peel extracts ranged from 6.35 μg/g FW (Liaofeng) to 14.52 μg/g FW (Changjiangyihao). The free proanthocyanidin B2 contents of flesh extracts ranged from 1.50 μg/g FW (Longcheng) to 5.77 μg/g FW (Huairou). The free proanthocyanidin B2 contents of peel extracts ranged from 2.95 μg/g FW (LD-121) to 9.59 μg/g FW (Huairou). The free proanthocyanidin C1 contents of flesh extracts ranged from 0.32 μg/g FW (Huairou) to 2.57 μg/g FW (LD-241). The free proanthocyanidin C1 contents of peel extracts ranged from 2.44 μg/g FW (Huairou) to 3.92 μg/g FW (Liaofeng). The flavanol contents of the peel extracts were higher than those of corresponding flesh extracts among all six kiwi berry varieties.

Three flavonols, i.e., quercetin-3-*O*-glucoside, quercetin-3-*O*-rutinoside, and quercetin-3-*O*-galactoside were only found in free fractions. The free quercetin-3-*O*-glucoside contents of flesh extracts ranged from 1.64 μg/g FW (Longcheng) to 4.79 μg/g FW (Liaofeng). The free quercetin-3-*O*-glucoside contents of peel extracts ranged from 4.62 μg/g FW (Longcheng) to 16.57 μg/g FW (LD-241). The free quercetin-3-*O*-rutinoside contents of flesh extracts ranged from 2.52 μg/g FW (LD-121) to 5.52 μg/g FW (Longcheng). The free quercetin-3-*O*-rutinoside contents of peel extracts ranged from 5.51 μg/g FW (LD-121) to 20.08 μg/g FW (Longcheng). The free quercetin-3-*O*-galactoside contents of flesh extracts ranged from 1.88 μg/g FW (Huairou) to 6.14 μg/g FW (Changjiangyihao). The free quercetin-3-*O*-galactoside contents of peel extracts ranged from 12.48 μg/g FW (LD-121) to 22.70 μg/g FW (Changjiangyihao). The flavonol contents of the peel extracts were higher than those of corresponding flesh extracts among all six kiwi berry varieties.

### Peroxyl Radical Scavenging Capacity of Kiwi Berry

**Figure 4:**
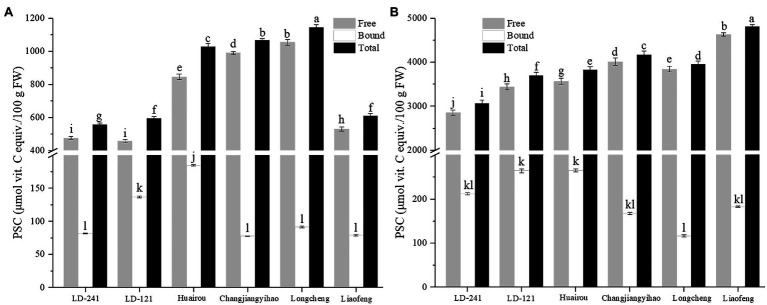
PSC values of flesh extracts **(A)** and peel extracts **(B)** of six kiwi berry varieties (mean ± SD; *n* = 3). Bars with different letters differ significantly (*p* < 0.05).

The PSC values of the flesh (Figure 4A) and peel (Figure 4B) extracts of the six kiwi berry varieties are shown in Figure 4. The free PSC values were significantly higher than those of the corresponding bound PSC values for both the flesh and peel extracts. As shown in Figure 4A, the free PSC values of flesh extracts ranged from 456.64 μmol VCE/100 g FW (LD-121) to 1053.60 μmol VCE/100 g FW (Longcheng), whereas the bound PSC values of flesh extracts ranged from 77.81 μmol VCE/100 g FW (Changjiangyihao) to 184.62 μmol VCE/100 g FW (Huairou). As shown in Figure 4B, the free PSC values of peel extracts ranged from 2855.71 μmol VCE/100 g FW (LD-241) to 4630.56 μmol VCE/100 g FW (Liaofeng), whereas the bound PSC values of flesh peel ranged from 116.40 μmol VCE/100 g FW (Longcheng) to 264.46 μmol VCE/100 g FW (Huairou). The PSC values of the peel were considerably higher than those of the flesh of all six kiwi berry varieties.

### Cellular Antioxidant Activity of Kiwi Berry

The CAA values of the flesh and peel extracts of the six kiwi berry varieties are shown in Table 4. Only the CAA of the free extract was determined because the CAA of the bound extract was too low to be determined. In the “no phosphate-buffered saline (PBS) wash” protocol, the CAA values of flesh extracts ranged from 49.73 μmol QE/100 g FW (LD-121) to 161.42 μmol QE/100 g FW (Longcheng). The CAA values of peel extracts ranged from 161.08 μmol QE/100 g FW (LD-241) to 297.34 μmol QE/100 g FW (Liaofeng). In the “PBS wash” protocol, the CAA values of flesh extracts ranged from 14.20 μmol QE/100 g FW (Changjiangyihao) to 33.58 μmol QE/100 g FW (LD-121). The CAA values of peel extracts ranged from 29.42 μmol QE/100 g FW (Changjiangyihao) to 71.37 μmol QE/100 g FW (LD-241). The CAA values of the peel extracts were higher than those of the corresponding flesh extracts in both the “no PBS” and “PBS wash” protocols.

**Table 4:** Cellular antioxidant activities of six kiwi berry varieties^a^.

Sample	CAA (μmol QE/100 g FW)			
	No PBS wash		PBS wash	
	Flesh	Peel	Flesh	Peel
LD-241	65.77 ± 3.45^d^	161.08 ± 2.35^e^	29.10 ± 2.67^b^	71.37 ± 2.23^a^
LD-121	49.73 ± 1.19^e^	188.41 ± 2.59^d^	33.58 ± 1.52^a^	56.48 ± 1.93^b^
Huairou	110.08 ± 3.86^c^	225.12 ± 4.38^c^	20.19 ± 1.47^c^	45.96 ± 1.48^b^
Changjiangyihao	126.85 ± 6.45^b^	230.75 ± 13.16^c^	14.20 ± 1.29^d^	29.42 ± 1.59^c^
Longcheng	161.42 ± 5.61^a^	249.23 ± 8.69^b^	14.82 ± 1.48^d^	45.40 ± 2.19^b^
Liaofeng	71.33 ± 4.12^d^	297.34 ± 12.06^a^	17.45 ± 1.27^cd^	29.78 ± 1.86^c^

a Results are expressed as mean ± SD (*n* = 3). Values of each column with no letters in common are significantly different (*p* < 0.05).

### Multivariate Correlation by Partial Least Squares Regression Among Phenolics Profile and Antioxidant Activities

**Figure 5:**
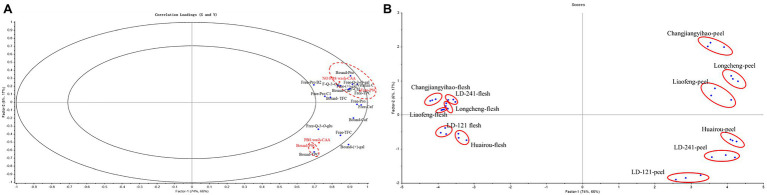
PLS plots of the correlations **(A)** and Scores **(B)** between phenolics profile and antioxidative activities in kiwi berry flesh and peel extracts. The antioxidative activities are in red font and phenolic profile are in blue font. Abbreviations of phenolic compounds: Pro, Protocatechuic acid; Caf, Caffeic acid; Chl, Chlorogenic acid; Qui, Quinic acid; (+)-gal (+)-gallocatechin; Pro B2, proanthocyanidin B2; Pro C1, proanthocyanidin C1; Q-3-*O*-glu quercetin-3-*O*-glucoside; Q-3-*O*-r quercetin-3-*O*-rutinoside; Q-3-*O*-gal, Quercetin-3-*O*-galactoside.

The multivariate correlation used the PLS regression models of various groups of the phenolics profile (e.g., phenolics, flavonoids, and vitamin C as well as individual phenolic compounds) with the antioxidant activity (PSC and CAA) of the six kiwi berry varieties as shown in Figure 5. Figure 5A shows the PLS plots of the kiwi berry extracts where 85.02% of the phenolics profile explained 93.79% of the variation in the antioxidant activities in two factors. As shown in Figure 5B, this PLS model could explain up to 90% of the data variability, and the clustering of different varieties of kiwi berry could be observed from all the data. Free-TPC, vitamin C, free-(+)-gallocatechin, and free-chlorogenic acid were positively correlated with free-PSC and no PBS wash-CAA values. Some phenolic monomers such as free-quercetin-3-*O*-galactoside and free-quercetin-3-*O*-rutinoside showed moderate contribution to free-PSC and no PBS wash-CAA values. Moreover, bound-TPC was positively correlated with the bound-PSC value. However, no phenolics profile correlated with the PBS wash-CAA values. Because of the PBS wash, the CAA value cannot reflect the total antioxidant activities of phenolics profile both inside and outside the cell.

## Discussion

### Total Phenolic Content of Kiwi Berry

Phenolics are strong antioxidants that are found in many fruits and vegetables (Liu, 2013). Phenolics reduce the risk of many chronic diseases, such as chemoresistance (Jing, 2008), and are anti-carcinogenic, anti-inflammatory, and protect against certain types of cancer (Kamiloglu, 2015). In this study, free-TPC is significantly higher than that of the corresponding bound-TPC, which is consistent with a previous study (Sun et al., 2002). Free phenolics reduce the oxidative stress response in cells and are rapidly released and absorbed in the stomach and small intestine (Chandrasekara and Shahidi, 2011). Bound phenolic extracts protect the digestive system and other cancers because the extracts are retained after digestion in the stomach and intestines and are released in the colon during the fermentation of colonic bacteria (Adom and Liu, 2002).

In this study, the free TPC of the peel extract (344.45 mg GAE/100 g FW) was higher than that of the free-TPC of the flesh extract (64.32 mg GAE/100 g FW). These results are consistent with a previous study (Latocha, 2015), in which the peel extract TPC (212.10 mg GAE/100 g FW) is higher than that of the flesh extract TPC (21.60 mg GAE/100 g FW). Moreover, bound-TPC of the peel extract (64.50 mg GAE/100 g FW) was higher than that of bound-TPC of the flesh extract (28.48 mg GAE/100 g FW). These results are consistent with previous results; the phenolic content in the peel is significantly higher than that in the flesh of all studied varieties of *A. arguta* (Latocha, 2015). The kiwi berry phenolic content is more than three times that of kiwifruit (Leontowicz, 2016). Therefore, the kiwi berry peel extract is an excellent source of phenolics and may play an important role in preventing chronic diseases in humans.

### Total Flavonoid Content of Kiwi Berry

Flavonoids are widely distributed phenolics with health-related properties that are based on their antioxidant activity (Benavente-Garcı´, 1997). Flavonoids prevent cardiovascular diseases and other cancers (Nayak et al., 2011; Romagnolo and Selmin, 2012). Previously, TFC has been determined using aluminum chloride (AlCl_3_; Froehlicher et al., 2009); however, due to anthocyanin interference, the detection of the flavonoid content was not accurate. Furthermore, the *p*-dimethylaminocinnamaldehyde method previously used has a low detection value (Gorinstein et al., 2009). Here, the SBC assay has been used to measure the total flavonoids, including flavonols, flavones, flavanols, flavanones, and anthocyanidins, which is an effective and comprehensive way to determine the TFC (He et al., 2008).

Flavonoids also exist in both free and bound form (Sheng et al., 2019). Similar to phenolics, the role of bound flavonoids cannot be ignored. The bound flavonoids may be absorbed by the intestinal membrane, which may be partially converted to glucuronic acid and sulfate (Liu, 2007). The TFC of kiwifruit (*Actinidia deliciosa*) has previously been reported to be 36.30 mg CE/100 g FW (Saeed et al., 2019), which is significantly lower than that of the kiwi berry (*A. arguta*) peel extract (114.82 mg CE/100 g FW) in this study. The flavonoid content in kiwi berry peel extracts was also higher than that in the corresponding flesh extracts. Moreover, kiwifruit had a lot of hair on the surface of the peel, rendering it unsuitable for consumption. In contrast, the kiwi berry skin is mostly smooth and without hair, which makes the whole fruit more suitable for direct consumption without removing the skin (Park et al., 2014). Therefore, the kiwi berry is a richer source of various phytochemicals than kiwifruit.

### Kiwi Berry Vitamin C Content

Vitamin C is considered the most important vitamin because it has significant antioxidant activity (Forastiere et al., 2000); for example, it protects cells from oxidative stress (Zhang et al., 2014) and has an important role in protecting the body against cardiovascular diseases (Padayatty et al., 2003). Moreover, vitamin C is one of the main antioxidants in body fluids; for example, it may relieve asthma symptoms in children (Kim et al., 2009). In this study, the vitamin C content was higher than that of other fruits, such as orange (51 mg/100 g FW), blackcurrant (52–122 mg/100 g FW), strawberry (29–48 mg/100 g FW; Lqbal et al., 2004), and traditional kiwifruit (Baranowska-Wójcik and Szwajgier, 2019). The vitamin C content of the purple star apple is higher in the peel extract than that in the flesh extract (Moo-Huchin et al., 2015).

### Phenolic Composition of Kiwi Berry

Protocatechuic acid, caffeic acid, chlorogenic acid, and quinic acid were the predominant phenolics in kiwi berry and could be detected in both free and bound extracts. Some studies indicated that protocatechuic acid had the potential of chemical protection, which inhibited chemical carcinogens *in vitro* and produced pro-apoptotic and anti-proliferative effects in different aspects (Tseng et al., 2000). Caffeic acid and chlorogenic acid were rich in ABTS and DPPH, which had the ability to scavenge free radicals (Gulcin, 2006). Due to its antioxidant constituents, quinic acid had played a role in preventing the development and progression of atherosclerotic disease (Hung et al., 2006). The results determined that the peel and flesh extracts of kiwi berry had a great significance. Comparatively, in peel extracts, the concentration of phenolic acids was more than that of corresponding flesh. Therefore, kiwi berry peel extracts may contribute significantly to endorse human health as a functional food.

The contents of (+)-gallocatechin, proanthocyanidin B2, and proanthocyanidin C1 were detected from the peel and flesh extracts of kiwi berry. Flavanols had *in vitro* antioxidant activity and potential anticancer ability (Rodriguez-Ramiro et al., 2011). The contents of quercetin-3-*O*-glucoside, quercetin-3-*O*-rutinoside, and quercetin-3-*O*-galactoside were also detected from the peel and flesh extracts of kiwi berry. Flavonols had a significant effect on reducing the risk of heart disease, especially atherosclerotic diseases (Ren et al., 2017).

Many studies illustrated that both intrinsic and extrinsic factors, such as genotypes, environmental variation, maturity, and post-harvest storage conditions, lead to differences in phenolic composition among the species (Pincemail et al., 2012; Ruiz et al., 2013). The different harvesting locations of kiwi berry were the direct cause of environmental differences. Therefore, further studies on the mechanisms of the influence of the environment on the antioxidants in kiwi berry are needed in the future.

The Pearson’s correlation analysis was used to determine the correlation between the TPCs, TFCs, vitamin C contents, and PSC values. The correlation of TPCs and PSC values was positive (flesh free extracts *R*^2^ = 0.924, *p* < 0.01; peel free extracts *R*^2^ = 0.976, *p* < 0.01; flesh bound extracts *R*^2^ = 0.890, *p* < 0.01; peel bound extracts *R*^2^ = 0.880, *p* < 0.01). Moreover, the correlation between vitamin C contents and PSC values was also positive (flesh extracts *R*^2^ = 0.870, *p* < 0.01; peel extracts *R*^2^ = 0.899, *p* < 0.01). However, no significant correlations were found between TFCs and PSC values. The correlation analysis suggested that phenolics and vitamin C made a significant contribution to the *in vitro* antioxidant activity.

### Peroxyl Radical Scavenging Capacity of Kiwi Berry

Antioxidant activity is important to human health, and it is also important to choose a favorable method to determine the antioxidant activity of bioactive compounds (Zang et al., 2021). In this study, the PSC assay (Adom and Liu, 2005) has been used to detect the total antioxidant activity among different parts of six kiwi berry varieties. Previously, the antioxidant activity has been mainly detected using the 2,2-diphenyl-1-picrylhydrazyl (DPPH), ferric reducing ability of plasma (FRAP), and 2,2-azino-bis(3-ethylbenzothiazoline-6-sulfonic acid) methods (Froehlicher et al., 2009; Lim et al., 2013). However, the FRAP measurement was conducted at pH 3.6, which is not a common condition in the human body (Zhang et al., 2021). The PSC assay was performed in a neutral environment, such as the human body (pH 7.4). Although chemically synthesized free radicals were used in the DPPH method, the PSC assay used the peroxyl radical, which naturally occurs in the human body (Zhang et al., 2021). According to a previous study, the PSC values of kiwi berry are higher than those of other fruits (Adom and Liu, 2005). In contrast to the bound extracts, free extract was a significant source of bioactive compounds.

A previous study showed that kiwi berry peel is an important source of bioactive compounds and is an important contribution to human health (Latocha, 2015). TPC, TFC, and vitamin C content of kiwi berry peel extracts were up to 10.77, 13.09, and 10.38 times richer than those of the corresponding flesh extracts, indicating the peel extracts had higher antioxidant activity than that of the flesh extracts, and the health benefits of the whole fruit depend on its flesh-to-peel ratio (Łata et al., 2005; Łata, 2007). These data suggested that kiwi berry peel might be a valuable material for the production of functional foods.

The Pearson’s correlation analysis was used to determine the correlation between the TPC, TFC, vitamin C content, and PSC values. The correlation between TPC and PSC values was positive (flesh free extracts *R*^2^ = 0.924, *p* < 0.01; peel free extracts *R*^2^ = 0.976, *p* < 0.01; flesh bound extracts *R*^2^ = 0.890, *p* < 0.01; and peel bound extracts *R*^2^ = 0.880, *p* < 0.01). Moreover, the correlation between vitamin C content and PSC values was also positive (flesh extracts *R*^2^ = 0.870, *p* < 0.01; peel extracts *R*^2^ = 0.899, *p* < 0.01). However, there were no significant correlations between the TFC and PSC values. The correlation analysis suggested that phenolics and vitamin C significantly contributed to the *in vitro* antioxidant activity.

### Cellular Antioxidant Activity of Kiwi Berry

The CAA assay represents a marked improvement over traditional chemical antioxidant activity assays, which simulate some *in vivo* cellular processes (Wolfe et al., 2008). The CAA values of the “PBS wash” protocol differed significantly from those of the “no PBS wash” protocol; the results are similar to those reported in other common fruits (Wolfe et al., 2008). The differences due to the PBS wash may influence the extracellular antioxidant capacity and reduce the overall cellular antioxidant capacity (Liu, 2007).

In this study, the kiwi berry CAA value is higher than that of blueberry (Wang et al., 2017), suggesting that kiwi berry extract may have better antioxidant activity. Meanwhile, the peel extract CAA values were higher than those of the corresponding flesh extracts, in agreement with the correlation of the PSC values and TPC. These results strongly suggested that the kiwi berry peel had strong antioxidant activity and might be a good source for producing functional foods.

The Pearson’s correlation analysis was used to determine the correlation between TPC, vitamin C content, and CAA values. The CAA values of the “no PBS wash” protocol were significantly correlated with the TPC (flesh extracts *R*^2^ = 0.909, *p* < 0.01; peel extracts *R*^2^ = 0.969, *p* < 0.01), and vitamin C content (flesh extracts *R*^2^ = 0.919, *p* < 0.01; peel extracts *R*^2^ = 0.940, *p* < 0.01). However, when cells were washed with PBS, the CAA values did not show any significant association with TPC and vitamin C content.

Natural antioxidants present in fruits have received much attention because of their assumed safety and potential nutritional and therapeutic value (Gao et al., 2020). Fruit peel is a powerful source of natural antioxidants, but it is usually wasted as part of the consumption and food industries. Fruit peels possess higher antioxidant compounds and antioxidant activity than fruit flesh (Wolfe et al., 2003; Ajila et al., 2007a). Therefore, fruit peel extracts, particularly those with high antioxidant activity, may be rich sources of antioxidants and deserve further study. According to these results, the high content of phenolics, vitamin C, and antioxidant activity of kiwi berries, especially peels, indicate that they may impart health benefits when consumed; opportunities for the food industry to develop ingredients for the formulation of functional food products are anticipated.

## Conclusion

Kiwi berry is considered a super food and one of the most nutritious fruits. This study aimed to assess and summarize the phenolics profile and antioxidant activities of six kiwi berry flesh and peel extracts, exploring their potential functional properties in foodstuffs. Ten phenolic monomers were analyzed qualitatively and quantitatively. The results showed that various groups of phenolics profiles are mainly free from both kiwi berry flesh and peel extracts. Moreover, kiwi berry peel possessed higher TPCs, TFCs, and VC content than kiwi berry flesh. Similarly, kiwi berry peel extracts have higher antioxidant contents. The multivariate correlation analysis showed that various groups of phenolics profiles are the main contributors to antioxidant activity. Therefore, kiwi berry peel may be utilized as a potential source of natural biologically active compounds. The potential application of kiwi berry peel extract in food, medicine, and cosmetics requires further research and discussion. In addition, the study provided detailed information on the phenolics profile and antioxidant activities of flesh and peel extracts, which will help researchers better understand the nutritional quality of kiwi berries and food manufacturers to develop tailor-made health products in China. In this field, further extensive research is still required to attract the food industrialists to add the value and develop kiwi-based food products.

## Resource Identification Initiative

Gallic acid (PubChem CID: 370), Catechin (PubChem CID: 9064), protocatechuic acid (PubChem CID: 72), caffeic acid (PubChem CID: 689043), chlorogenic acid (PubChem CID: 1794427), quinic acid (PubChem CID: 6508) (+)-gallocatechin (PubChem CID: 65084), proanthocyanidin B2 (PubChem CID: 122738), proanthocyanidin C1 (PubChem CID: 169853), quercetin-3-*O*-glucoside (PubChem CID: 5280804), quercetin-3-*O*-rutinoside (PubChem CID: 5280805), quercetin-3-*O*-galactoside (PubChem CID:5281643), and quercetin (PubChem CID: 5280343).

## Data Availability Statement

The original contributions presented in the study are included in the article/Supplementary Material, further inquiries can be directed to the corresponding authors.

## Author Contributions

JZ and BL designed the experiments. JZ and JT interpreted the results. JZ wrote the paper. JZ and NG participated in the data mining. GX, SC, and XS helped in the collection of kiwi berry materials. CS and CL helped to improve the manuscript. All authors contributed to the article and approved the submitted version.

### Conflict of Interest

The authors declare that the research was conducted in the absence of any commercial or financial relationships that could be construed as a potential conflict of interest.
