# Supplementary material for: Phenolics Profile and Antioxidant Activity Analysis of Kiwi Berry (Actinidia arguta) Flesh and Peel Extracts From Four Regions in China
Source: Front Plant Sci. 2021 Jul 1;12:689038. doi: 10.3389/fpls.2021.689038 (PMC8282361; doi:10.3389/fpls.2021.689038)
Supplement: Supplementary file 1 [file Data_Sheet_1.docx]

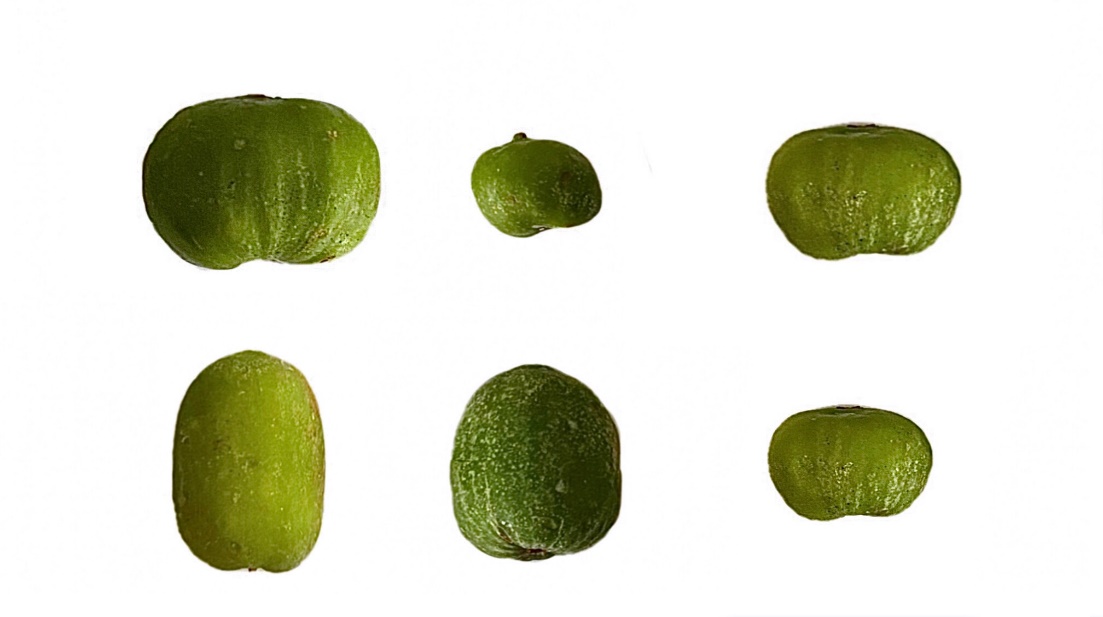


LD-241

LD-121

Huairou

Changjiangyihao

Longcheng

Liaofeng


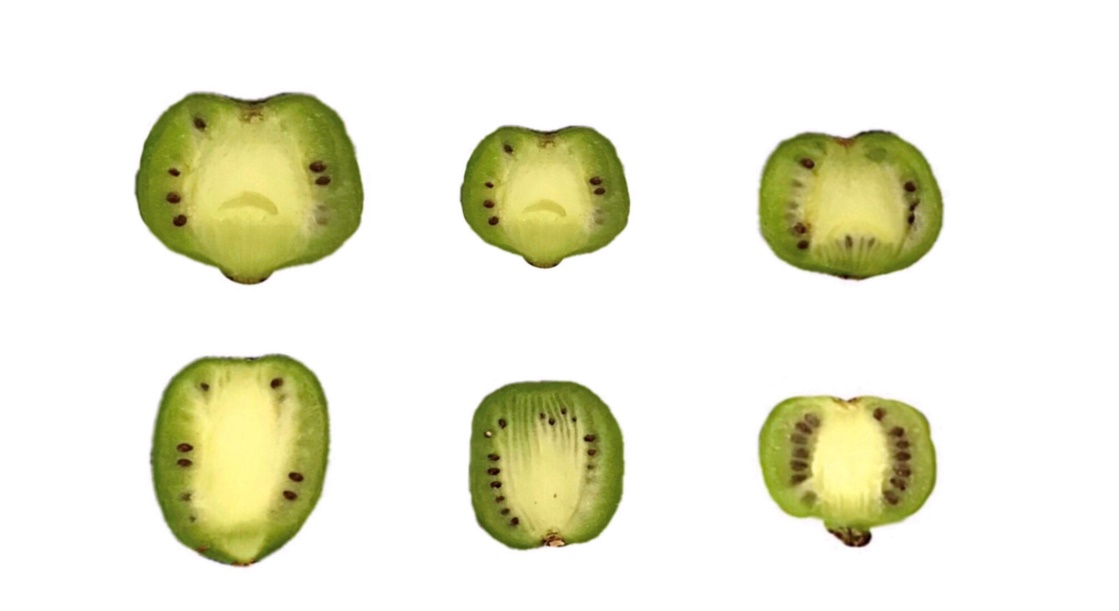


LD-241

LD-121

Huairou

Changjiangyihao

Longcheng

Liaofeng


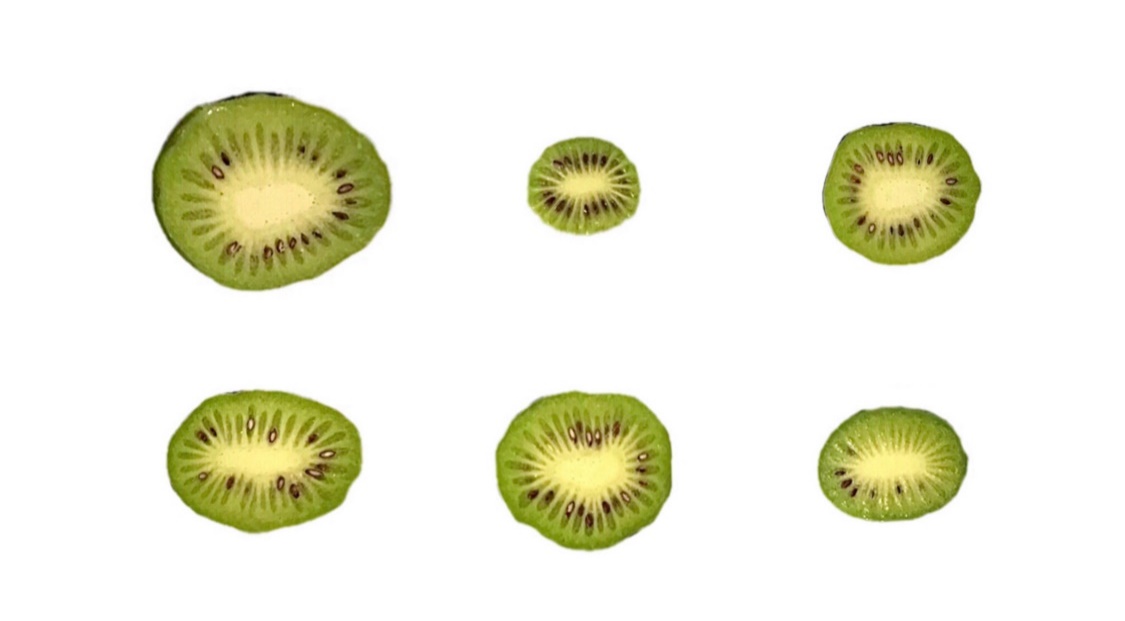


LD-241

LD-121

Huairou

Changjiangyihao

Longcheng

Liaofeng

**Supplementary Figure 1.** The photographs of six kiwi berry varieties.
